# Theory of Structural and Secondary Relaxation in Amorphous Drugs under Compression

**DOI:** 10.3390/pharmaceutics12020177

**Published:** 2020-02-19

**Authors:** Anh D. Phan, Katsunori Wakabayashi

**Affiliations:** 1 Faculty of Materials Science and Engineering, Phenikaa Institute for Advanced Study, Phenikaa University, Hanoi 12116, Vietnam; 2 Faculty of Information Technology, Artificial Intelligence Laboratory, Phenikaa University, Hanoi 12116, Vietnam; 3 Department of Nanotechnology for Sustainable Energy, School of Science and Technology, Kwansei Gakuin University, Sanda, Hyogo 669-1337, Japan; waka@kwansei.ac.jp

**Keywords:** compression effects, amorphous drugs, structural relaxation, secondary relaxation, glass transition, molecular dynamics, indomethacin, curcumin, glibenclamide

## Abstract

Compression effects on alpha and beta relaxation process of amorphous drugs are theoretically investigated by developing the elastically collective nonlinear Langevin equation theory. We describe the structural relaxation as a coupling between local and nonlocal activated process. Meanwhile, the secondary beta process is mainly governed by the nearest-neighbor interactions of a molecule. This assumption implies the beta relaxation acts as a precursor of the alpha relaxation. When external pressure is applied, a small displacement of a molecule is additionally exerted by a pressure-induced mechanical work in the dynamic free energy, which quantifies interactions between a molecule with its nearest neighbors. The local dynamics has more restriction and it induces stronger effects of collective motions on single-molecule dynamics. Thus, the alpha and beta relaxation times are significantly slowed down with increasing compression. We apply this approach to determine the temperature and pressure dependence of the alpha and beta relaxation time for curcumin, glibenclamide, and indomethacin, and compare numerical results with prior experimental studies. Both qualitative and quantitative agreement between theoretical calculations and experiments validate our assumptions and reveal their limitations. Our approach would pave the way for the development of the drug formulation process.

## 1. Introduction

Recently, much attention has been devoted to understand molecular dynamics of amorphous drugs during vitrification due to the enhancement of solubility and bioavailability compared to their crystalline forms [1,2,3,4]. By cooling molten materials with a fast rate, the structure of these pharmaceutical products becomes disordered and has amorphous form. During vitrification, molecules in amorphous materials undergo different relaxation processes including primary (structural/alpha) and secondary (beta) relaxation. These relaxations are strongly temperature-dependent and external pressures [5,6,7,8]. Thus, compression effects in the usage of compressors to generate tablets and thermal variations in many phases of pharmaceutical manufacturing and storage directly alter drug quantity [1,2], particularly the recrystallization of drugs. It is necessary to deeply understand glassy states and molecular mobility of amorphous drugs under compression conditions. The knowledge would facilitate and accelerate design and formulation process of pharmaceutical products having desired properties [1,2].

Apart from experiments, some theoretical approaches can be employed to investigate the molecular dynamics of amorphous systems. Although simulations can help us to understand how molecules interact with each other, it is impossible to access the experimental observation timescale (100 s). The simulation timescale of relaxation process is less than $10^{7}$ ps. Meanwhile, the Elastically Collective Nonlinear Langevin Equation (ECNLE) theory has been developed to quantitatively determine the temperature dependence of the structural relaxation time at ambient pressure [9,10,11,12,13,14,15] from 1 ps to more than 100 s. The ECNLE theory describes an amorphous material as a fluid of spherical molecular particles. The mean time for a particle to escape from its particle cage formed by the nearest neighboring molecules is the alpha relaxation time [9,10,11,12,13,14,15]. The relaxation is significantly slowed down with an increase of density. To compare theoretical calculations with experimental data, Phan and his coworkers [14,15] used the thermal expansion process to propose a density-to-temperature conversion (a thermal mapping) from the averaged particle density to temperature. This predictive approach has successfully explained the temperature-dependent molecular dynamics in single- and multi-component amorphous drugs [14,15].

In this paper, we develop the ECNLE theory to study the pressure and temperature dependence of alpha and beta relaxation time. We clearly reveal nature of these relaxation processes and their correlations. Our understandings are consistent with prior works. To validate the new developments more, we quantitatively compare theoretical calculations with experimental data of curcumin, glibenclamide, and indomethacin in previous studies [16,17,18].

## 2. Structural Relaxation Time of Amorphous Drugs under Pressure

### 2.1. Local and Collective Dynamics

**Figure 1:**
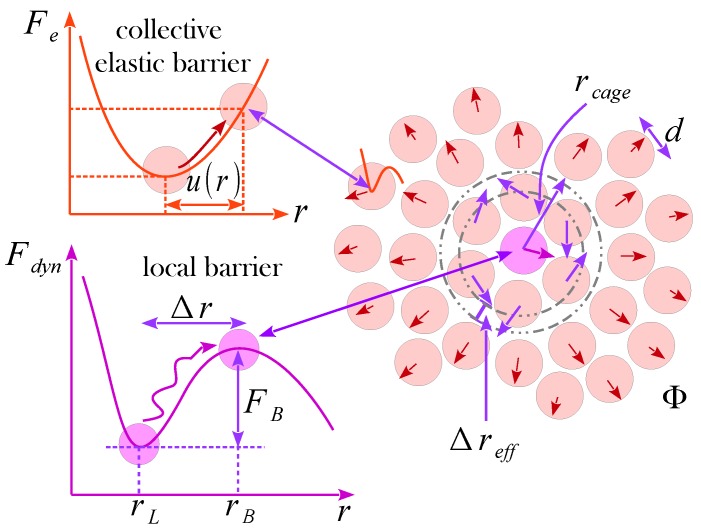
Schematic of the structural relaxation process including local cage-scale dynamics coupled to long-range collective elastic harmonic motions. Key length scales and a barrier for the cage-scale hopping of a tagged particle are indicated in the dynamic free energy profile. The jump distance is used to set the amplitude of the displacement field outside the particle cage.

The activation events in amorphous drugs are theoretically investigated using a hard-sphere model associated with ECNLE theory [9,10,11,12,13,14,15,19,20]. We describe amorphous drugs as hard-sphere fluid characterized by a particle diameter, *d*, and the number of particles per volume, ρ, as shown in Figure 1. Under an external pressure *P*, motions of a tagged particle are governed by its interactions with nearest neighbors and pressure-induced constraint. Thus, the free dynamic energy at temperature *T* of the tagged particle in ECNLE theory, which captures effects of these governing factors, is modified to be

(1) $$\begin{array}{ccc} \frac{F_{dyn}\left(r\right)}{k_{B}T} & = & \int_{0}^{\infty }dq\frac{q^{2}d^{3}{\left[S(q)-1\right]}^{2}}{12\pi \Phi \left[1+S(q)\right]}\exp\left[-\frac{q^{2}r^{2}\left(S\left(q\right)+1\right)}{6S(q)}\right]\\ & - & 3\ln\frac{r}{d}+\frac{P}{k_{B}T/d^{3}}\frac{r}{d}, \end{array}$$

where $k_{B}$ is Boltzmann constant, *r* is the displacement of the particle, S(q) is the static structure factor, *q* is the wavevector, and $\Phi =\rho \pi d^{3}/6$ is the volume fraction. Effects of rotational motions are not taken into account in Equation (1). The knowledge of S(q) and the radial distribution function g(r) for hard-sphere fluids can be calculated using the Persus–Yevick (PY) theory [21] for a hard-sphere fluid. According to the PY theory, the direct correlation function is $C\left(q\right)=\left[S(q)-1\right]/\rho S\left(q\right)$, while the Fourier transform of C(q) is [21]

(2) $$\begin{array}{ccc} C(r) & = & -\frac{{\left(1+2\Phi \right)}^{2}}{{\left(1-\Phi \right)}^{4}}+\frac{6\Phi {\left(1+\Phi /2\right)}^{2}}{{\left(1-\Phi \right)}^{4}}\frac{r}{d}\\ & - & \frac{\Phi {\left(1+2\Phi \right)}^{2}}{2{\left(1-\Phi \right)}^{4}}{\left(\frac{r}{d}\right)}^{3}\;\mathrm{for}\;r\leq d \end{array}$$

(3) $$\begin{array}{ccc} C(r) & = & 0\;\mathrm{for}\;r>d. \end{array}$$

The first term of Equation (1) is structure-dependent since it is derived from inherently interacting the coordination particles. The second term is due to the ideal fluid state, which is structure-independent and causes particle delocalization. Meanwhile, suppose that the displacement of particles is very small, and the third term comes from the mechanical work done by pressure acting on a volume $\Delta V\left(r\right)\approx rd^{2}$. An additional energy in the dynamic free energy caused by applying pressure is $P\Delta V\left(r\right)\approx Prd^{2}$.

Physical quantities of local dynamics are obtained using the free energy profile as depicted in Figure 1. In low-density systems, $F_{dyn}\left(r\right)$ decreases with an increase of *r* and particles are not dynamically arrested [19,20,21]. In dense systems, a free-energy barrier emerges and one observes onset of the dynamical confinement of particles (motions of the tagged particle within a particle cage formed by its neighbors). The particle cage radius, $r_{cage}$, is approximately determined as a position of the first minimum of g(r). Numerical results of Equation (1) give the local minimum (localization length $r_{L}$) and maximum (the barrier position $r_{B}$) of the dynamic free energy. From these, we can calculate a jump distance and a local energy-barrier height defined by $\Delta r=r_{B}-r_{L}$ and $F_{B}=F_{dyn}\left(r_{B}\right)-F_{dyn}\left(r_{L}\right)$, respectively.

When a particle escapes from its cage, the diffusion is affected by not only nearest-neighbor interactions, but also cooperative motions of surrounding particles. The rearrangement of particles in the first shell causes the cage expansion on the surface and propagates outward radially a harmonic displacement field u(r). In bulk systems, one can find an analytical form of the distortion field by Lifshitz’s continuum mechanics analysis [22], which is

(4) $$\begin{array}{c} \left(K_{B}+\frac{G}{3}\right)\nabla \left(\nabla .u\right)+G\nabla^{2}u=0, \end{array}$$

where $K_{B}$ is the bulk modulus and *G* is the shear modulus. Since u(r) is purely radial in bulk systems, Equation (4) becomes $\nabla^{2}u=0$ and its solution is

(5) $$\begin{array}{c} u\left(r\right)=\Delta r_{eff}\frac{r_{cage}^{2}}{r^{2}},\;r\geq r_{cage}, \end{array}$$

where $\Delta r_{eff}$ is the amplitude of the cage expansion at the surface [10,11] calculated by

(6) $$\begin{array}{c} \Delta r_{eff}=\frac{3}{r_{cage}^{3}}\left[\frac{r_{cage}^{2}\Delta r^{2}}{32}-\frac{r_{cage}\Delta r^{3}}{192}+\frac{\Delta r^{4}}{3072}\right]. \end{array}$$

Since $\Delta r_{eff}\ll r_{cage}$ is relatively small, collective vibrations of particles beyond the particle cage can be viewed as harmonic oscillations with a spring constant at $K_{0}={\left|\partial^{2}F_{dyn}\left(r\right)/\partial r^{2}\right|}_{r=r_{L}}$. The total elastic energies of these oscillators can be used to determine effects of collective motions on the relaxation event. The elastic energy of a particle at a distance *r* from the center of a particle cage is $K_{0}u^{2}\left(r\right)/2$. Because the number of particles at a distance between *r* and r+dr is $\rho g\left(r\right)4\pi r^{2}dr$, the net elastic barrier is

(7) $$\begin{array}{c} F_{e}=4\pi \rho \int_{r_{cage}}^{\infty }drr^{2}g\left(r\right)K_{0}\frac{u^{2}\left(r\right)}{2}. \end{array}$$

For $r\geq r_{cage}$, g(r)≈1. The calculation suggests that effects of collective dynamics of particles are distinct but strongly related to those of local dynamics.

### 2.2. Relaxation Process

Chemical and biological complexities, conformational configuration, and chain connectivity have been found to cause non-universal coupling between local and non-local dynamics [15]. To address this issue, we introduced an adjustable parameter $a_{c}$ to adjust the relative role of the collective elastic barrier on the glass transition as $F_{e}\to a_{c}^{2}F_{e}$ [15]. The introduction of $a_{c}$ is based on the hypothesis that the amplitude of cage expansion is sensitive to subnanometer chemical (conformational) complexities, which are coarse-grained over in the hard sphere model. According to Kramer’s theory, the structural (alpha) relaxation time is

(8) $$\begin{array}{c} \frac{\tau_{\alpha }}{\tau_{s}}=1+\frac{2\pi }{\sqrt{K_{0}K_{B}}}\frac{k_{B}T}{d^{2}}\exp\left(\frac{F_{B}+a_{c}^{2}F_{e}}{k_{B}T}\right), \end{array}$$

where $K_{B}$=${\left|\partial^{2}F_{dyn}\left(r\right)/\partial r^{2}\right|}_{r=r_{B}}$ is absolute curvatures at the barrier position and $\tau_{s}$ is a short time scale of relaxation. The explicit expression of $\tau_{s}$ is [10,11]

(9) $$\begin{array}{c} \tau_{s}=g^{2}\left(d\right)\tau_{E}\left[1+\frac{1}{36\pi \Phi }\int_{0}^{\infty }dq\frac{q^{2}{\left(S\left(q\right)-1\right)}^{2}}{S(q)+b(q)}\right], \end{array}$$

where $\tau_{E}$ is the Enskog time scale, $b\left(q\right)=1/\left[1-j_{0}\left(q\right)+2j_{2}\left(q\right)\right]$, and $j_{n}\left(x\right)$ is the spherical Bessel function of order *n*. In prior works [9,10,11,14,15], $\tau_{E}\approx 10^{-13}$ s is used for amorphous materials including thermal liquids, polymers, and amorphous drugs. By using this approach, we have accurately and simultaneously predicted the temperature dependence of $\tau_{\alpha }$, the glass transition temperature, and the dynamic fragility for 22 amorphous drugs and polymers [15].

Meanwhile, the beta relaxation process is typically attributed to either intramolecular motions or fast single molecule dynamics known as the Johari–Goldstein (JG) process. In the ENCLE theory, the latter process is viewed as the local dynamics of a single particle within its particle cage. This assumption implies that effects of collective motions of molecules outside the cage on the JG relaxation can be completely ignored. Consequently, to calculate the JG beta relaxation time, only local barrier $F_{B}$ is taken into account in the Kramer’s theory. The beta relaxation time is

(10) $$\begin{array}{c} \frac{\tau_{\beta }}{\tau_{s}}=1+\frac{2\pi }{\sqrt{K_{0}K_{B}}}\frac{k_{B}T}{d^{2}}e^{F_{B}/k_{B}T}. \end{array}$$

In recent works [14,15], we proposed a density-to-temperature conversion (thermal mapping) based on the thermal expansion during heating to determine the temperature dependence of the structural relaxation time. The thermal mapping is [14,15]

(11) $$\begin{array}{c} T\approx T_{0}-\frac{\Phi -\Phi_{0}}{\beta \Phi_{0}}. \end{array}$$

where $\beta \approx 12\times 10^{-4}$$K^{-1}$ is the common volume thermal expansion coefficient for all organic materials and $\Phi_{0}\approx 0.5$ is the characteristic volume fraction. From the hard-sphere calculations, we adjust $T_{0}$ and $a_{c}$ to obtain the best fit between theory and experiment for the temperature dependence of $\tau_{\alpha }$. From this, we can determine the relative importance between local and collective motions on the glassy dynamics of amorphous drugs.

## 3. Results and Discussion

**Figure 2:**
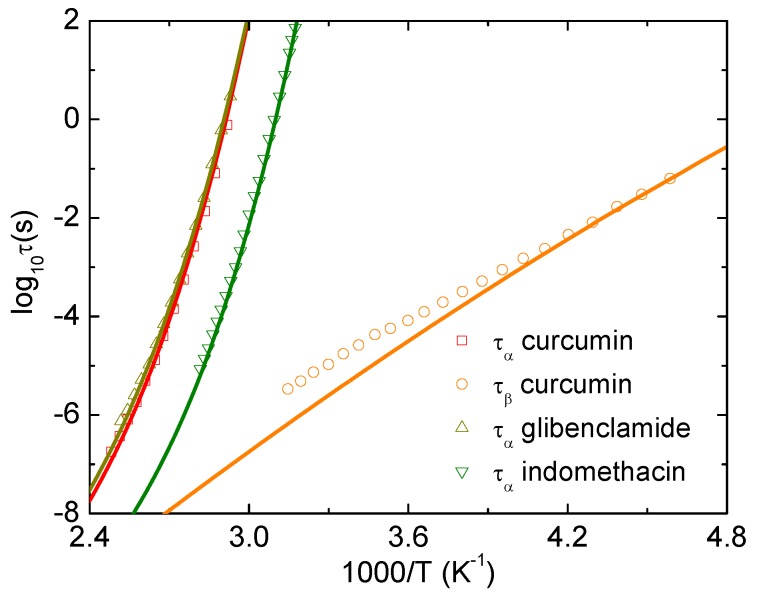
Logarithm of alpha and beta relaxation time of curcumin, glibenclamide, and indomethacin as a function of 1000/T at ambient pressure. Open points are experimental data in Refs. [16,17,18] and solid curves correspond to our ECNLE calculations.

We use Equations (8)–(11) to theoretically calculate the temperature dependence of the alpha and beta relaxation time of curcumin, glibenclamide, and indomethacin. Figure 2 shows theoretical and experimental temperature dependence of $\log_{10}\tau_{\alpha }$ and $\log_{10}\tau_{\beta }$ under atmospheric pressure. The parameters used in calculations are $T_{0}=518.6$ *K* and $a_{c}=1$ for curcumin, and $T_{0}=525$ *K* and $a_{c}=0.9$ for glibenclamide, and $T_{0}=476$ *K* and $a_{c}=1.5$ for indomethacin. The value of $a_{c}$ acquires material specificity. The ENCLE calculations agree quantitatively well with experimental data [16,17,18]. Particularly, the agreement for curcumin is obtained without any adjustable parameter ($a_{c}=1$ implies a regular or uncorrected local-nonlocal-dynamics relationship). This quantitative accordance identifies a strong correlation between the alpha and beta process of curcumin. These calculations indicate that intramolecular motions play a minor role in these two types of relaxation and clearly confirms a nature of the beta process, which is the JG relaxation.

Our ECNLE approach is a minimalist model to calculate properties of glassy dynamics and compare them to experimental data. Based on good accordance between theory and experiment, we can reveal underlying physical mechanisms. Within ECNLE theory, effects of the chemical structures of organic glasses on their dynamics are encoded in two parameters: the local-nonlocal coupling $a_{c}$ and the characteristic temperature $T_{0}$. Although these simplified assumptions have worked well with the alpha relaxation, theoretical predictions of the secondary relaxation may deviate from experiments. This is because understanding the secondary dynamics is a challenging problem. However, the deviation can be used to determine contribution of chemical and biological interactions to the beta and gamma relaxation. A good quantitative agreement between theory and experiment suggests a possibility of ignoring effects of chemical structures and this is the case of curcumin as shown in Figure 2.

Applying an external pressure to the amorphous drug localizes more the single-molecular dynamics within its cage and significantly slows-down molecular mobility. In Equation (1), the pressure enters the dynamic free energy in units of $k_{B}T/d^{3}$, enlarging the local barrier height $F_{B}$ and the jump distance Δr. These behaviors are also equivalent to rising the effective volume fraction Φ at ambient pressure. It suggests that our treatment of Equation (1) is the same as considering hard-sphere fluids with higher molecular packing. An increase of the jump distance leads to a growth of the collective barrier since $F_{e}∼K_{0}\Delta r^{4}$. Suppose that the correlation between local and collective dynamics remains unchanged and the thermal mapping in Equation (11) is also unaffected under compression. The growth of both barriers leads to an increase of $\tau_{\alpha }$ and $\tau_{\beta }$ with increasing pressure. To compare our numerical results to experiments, we define the glass transition pressure, $P_{g}$, where $\tau_{\alpha }\left(P_{g}\right)=100$ s for curcumin [16] and $\tau_{\alpha }\left(P_{g}\right)=1$ s for glibenclamide [17] at a given temperature and then normalize the pressure with $P_{g}$.

**Figure 3:**
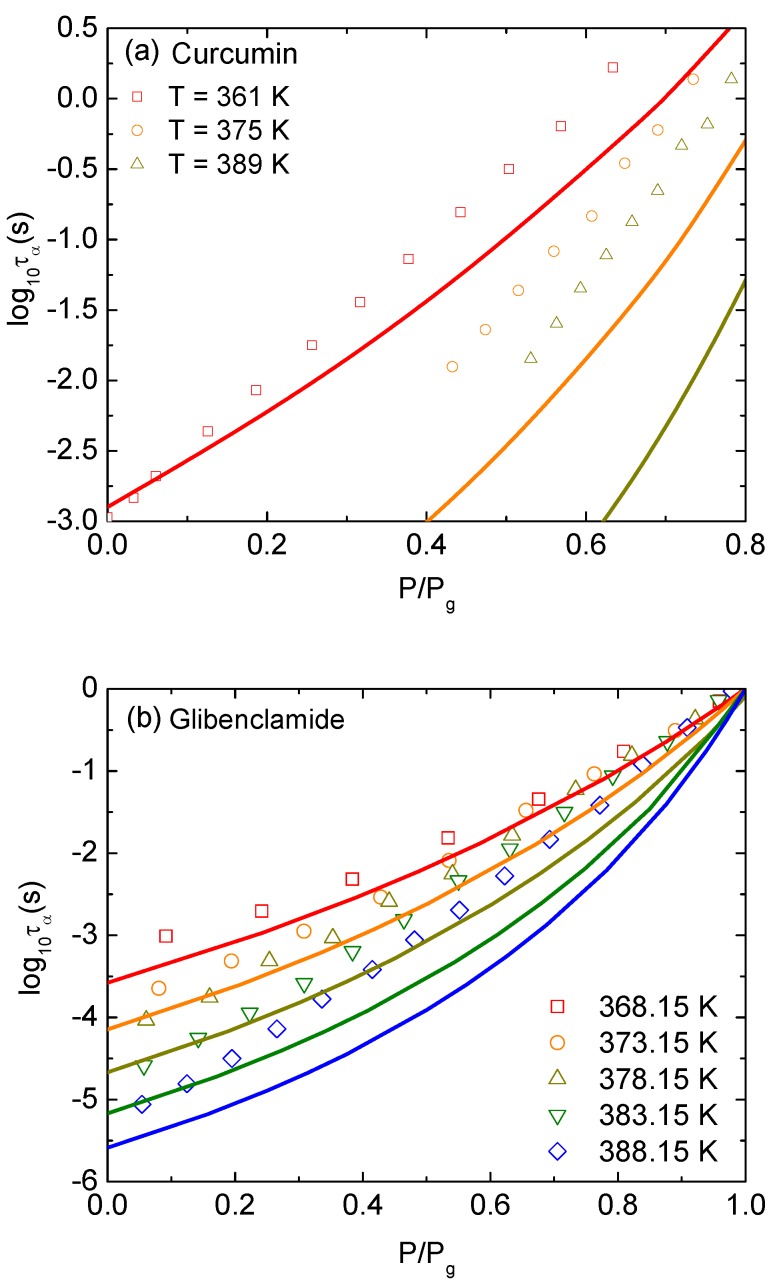
Logarithm of structural relaxation time at isothermal condition of (**a**) curcumin at T=361, 375, and 389 *K* versus pressure normalized the pressure at $P_{g}$ defined by $\tau_{\alpha }\left(P_{g}\right)=100$ s, and (**b**) glibenclamide at T=368.15, 373.15, 378.15, 383.15, and 388.15 *K* versus pressure normalized the pressure at $P_{g}$ defined by $\tau_{\alpha }\left(P_{g}\right)=1$ s. Open points are experimental data in Refs. [16,17] and solid curves correspond to our ECNLE calculations.

Figure 3a shows experimental data in Ref. [16] and theoretical calculations for $\tau_{\alpha }$ versus normalized pressure of curcumin at T=361, 375, and 389 *K*. Over a wide range of timescale, the growths of $\tau_{\alpha }\left(T\right)$ with increasing compression predicted by ECNLE theory are not close to the experimental counterparts. Experimental data points are higher than theoretical curves. Accordance between theory and experiment becomes better at low temperatures. One can see the same behaviors exhibiting in Figure 3b when the pressure dependence of $\tau_{\alpha }$ for glibenclamide at isothermal condition is determined.

A main reason for the deviation in Figure 3 to appear is that curcumin has a very strong H-bonded active ingredient, and glibenclamide has a large fluctuation of charge distribution. The electrostatic forces between molecules affect the relaxation processes and can somehow invalidate the hard sphere model in ECNLE theory. The forces may be weak in room conditions since the good agreement between the ECNLE calculations and experiment in Figure 2 confirms it. However, at much higher pressures, charged atoms are enforced to be closer and roles of the electrostatic energy on the structural arrangement and the glass transition becomes more important. Thus, the quantitative agreement in Figure 3 can be improved by considering intermolecular forces. For a given interaction potential, one can employ the standard reference interaction site model (RISM) [21] to generate the radial distribution function g(r) and the static structure factor S(q). Then, these quantities are simply inserted into Equation (1) to calculate relaxation times as described in the previous section. Since we do not know parameters of the intermolecular interactions, numerical results are controlled by 3–4 variables/adjustable parameters including $T_{0}$, $a_{c}$, and the length scale and amplitude of interaction energy. The problem is interesting but complicated.

Another main reason is that the molecular size is supposed to be unchanged with pressure variation. To capture compression effects on the molecular size and simultaneously obtain better agreement with experiment, we consider the diameter *d* as an adjustable parameter which is pressure-dependent. This assumption is quite reasonable since the external pressure changes steric repulsion between molecules as discussed above. It is well-known that the steric effects have a strong influence on the molecular conformation and volume. Thus, under the isobaric process, we can find a theoretical value of *P* to obtain a good agreement with experimental data. The equality between theoretical and experimental pressure gives us the particle diameter in nanometer units.

**Figure 4:**
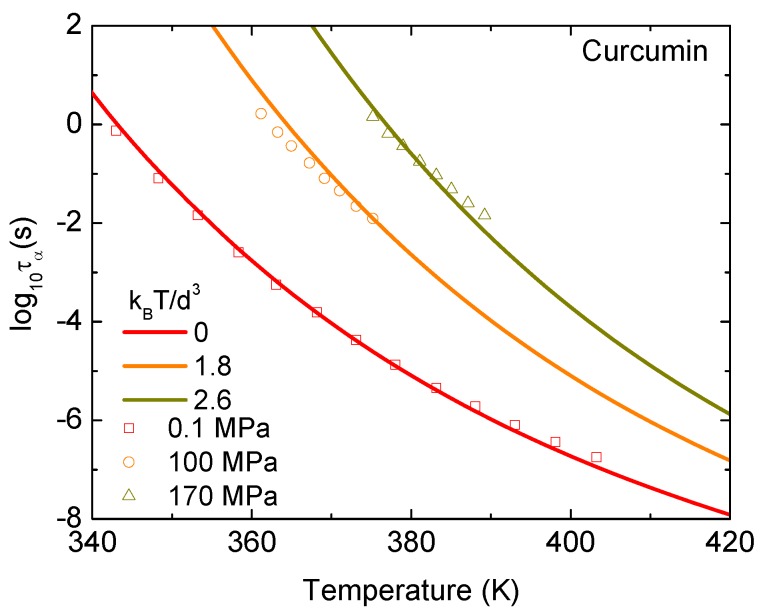
Logarithm of alpha relaxation time of curcumin as a function of temperature at P=0.1, 100, and 170 MPa. Open points are experimental data in Ref. [16] and solid curves correspond to our ECNLE calculations.

Figure 4 shows experimental data and theoretical calculations for the temperature dependence of $\tau_{\alpha }$ of curcumin at fixed pressures. Theoretical pressures are set to be 0, 1.8, and 2.6 $k_{B}T/d^{3}$, while experimental pressures are 0.1, 100, and 170 MPa. The accordance between theory and experiment suggests that 1.8 $k_{B}T/d^{3}$ = 100 MPa and 2.6 $k_{B}T/d^{3}$ = 170 MPa. One can see that the molecular/particle volume expands in a linear manner with *T* and/or $d∼T^{-1/3}$ under high isobaric conditions. We can determine d≈0.449 nm at T=365 *K* for P= 100 MPa and 0.429 nm at T=375 *K* for P= 170 MPa. These values are the same order of Kuhn segment size in polymeric materials [23]. The rule of the thermal expansion of the volume per a molecule is invalid at low temperatures.

**Figure 5:**
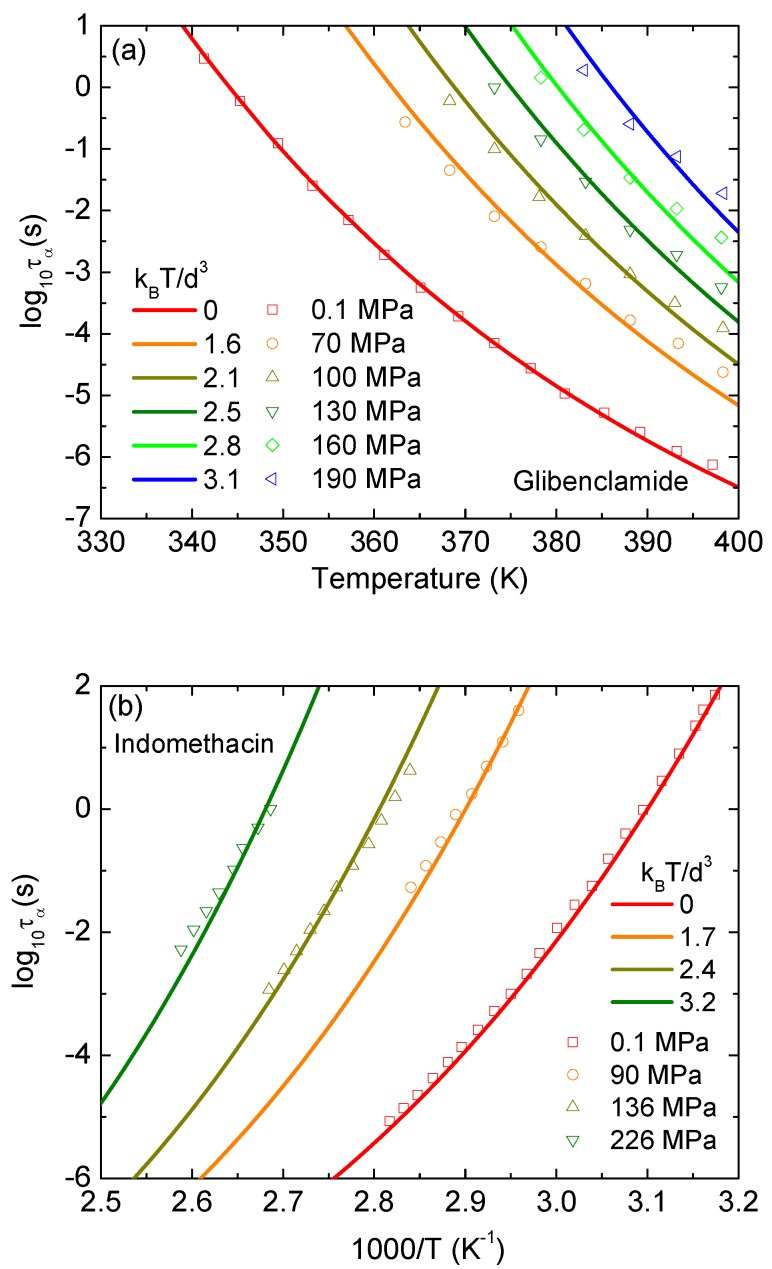
Logarithm of structural relaxation time of (**a**) glibenclamide as a function of temperature, and (**b**) indomethacin as a function of 1000/*T*. Open points are experimental data in Refs. [17,18], and solid curves correspond to our ECNLE calculations.

We implement the same analysis to glibenclamide and indomethacin, and show results in Figure 5. All behaviors seen in Figure 4 are repeated in Figure 5. Again, theoretical curves perfectly overlap the corresponding experimental curves at low pressures and slight deviations occur at higher pressures. The theoretical predictions are relatively steeper than the corresponding experimental data. The deviation is unavoidable since our model provides a minimalist approach and may have some missing physics.

**Figure 6:**
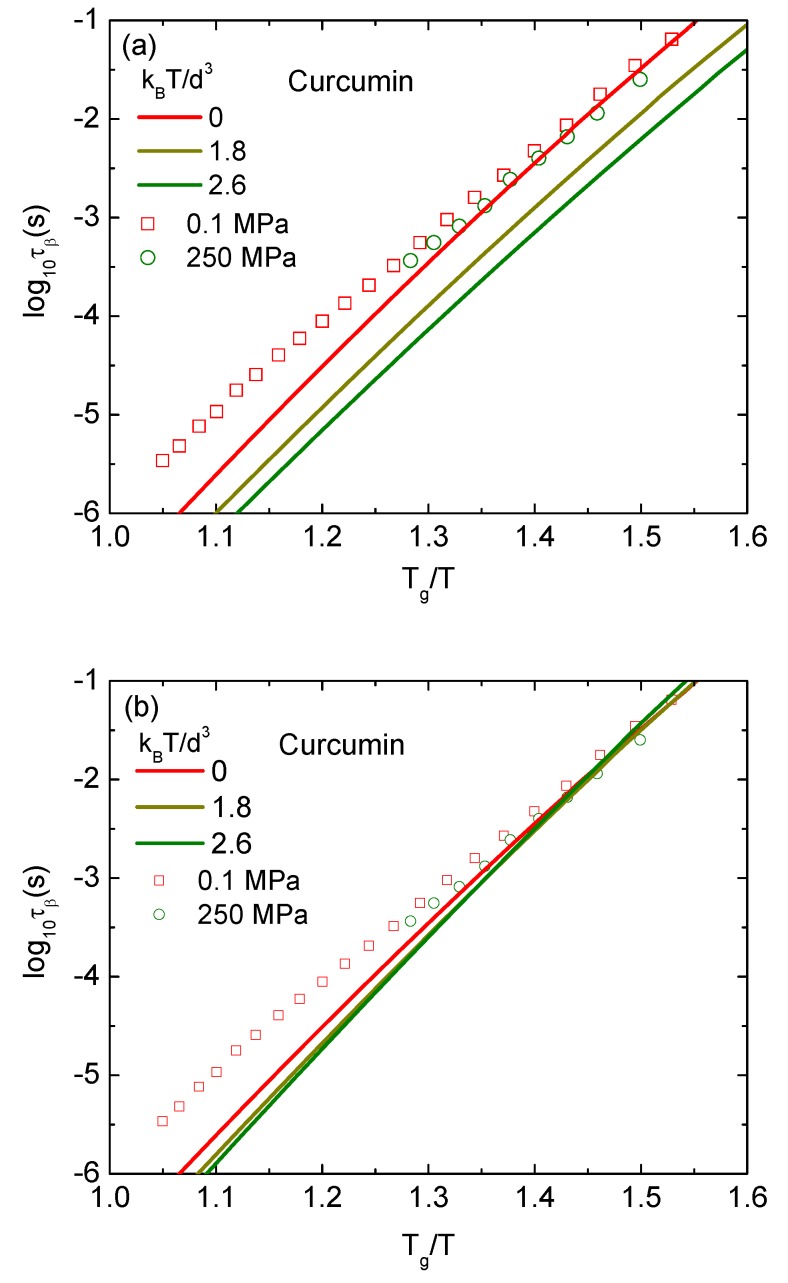
Logarithm of secondary (beta) relaxation time of curcumin as a function of inversely normalized temperature at different pressures. Open points are experimental data in Ref. [16] and solid curves correspond to our ECNLE calculations for (**a**) no change in the thermal mapping and (**b**) adjusted values of $\beta_{c}=4.72\times 10^{-4}$ and $4.82\times 10^{-4}$$K^{-1}$ for P = 1.8 and 2.6 $k_{B}T/d^{3}$, respectively.

The temperature dependence of the beta relaxation times at different pressures calculated by the ECNLE theory are contrasted with experimental data in Figure 6. In these calculations, the thermal mapping in Equation (11) used for the structural relaxation time is now applied to the beta process without any modification. At ambient pressure, our numerical results can describe the experiments quantitatively. In higher compression conditions, although experimental study in Ref. [16] reveals the pressure independence of the beta relaxation time, theoretical calculations in Figure 6a indicate the pressure dependence of $\tau_{\beta }$. The beta relaxation time as a function of $T_{g}/T$ is lowered by the same order of magnitude as increasing pressure. As seen in Figure 6a, theoretical curves corresponding to P=0, 1.8 and 2.6 $k_{B}T/d^{3}$ are approximately “parallel” to each other. Note that P=1.8 and 2.6 $k_{B}T/d^{3}$ are equivalent to 100 and 170 MPa, respectively. This finding suggests that the thermal mapping for the beta process has to be modified.

In the JG beta relaxation, a single particle moves freely fast within its particle cage. The motion is similar to vibration of an atom around its equilibrium position in a crystal lattice. Thus, the thermal expansion coefficient β in the thermal mapping for the beta process cannot be considered as in an amorphous/disordered state. Its value has to be estimated as in the crystal state. Because of different molecular mobility, the expansion coefficients of the glass-forming liquids $\beta_{g}$ are typically 2–4 times larger than those of crystal counterparts $\beta_{c}$ [24]. By reducing $\beta_{c}$ to $4.72\times 10^{-4}$ and $4.82\times 10^{-4}$$K^{-1}$ for P=1.8 and 2.6 $k_{B}T/d^{3}$, respectively, we can see the pressure insensitivity of $\tau_{\beta }\left(T\right)$ in Figure 6b, which is consistent with experiments in Ref. [16]. Our adjusted values of $\beta_{c}$ are relatively reasonable. However, from a condensed matter physics point of view, an increase of external pressure induces a decrease of $\beta_{c}$ if nothing changes in size and chemical/biological conformation since molecular dynamics have more restrictions. A little rise of $\beta_{c}$ in our calculations reveals a complicated competition between a decrease in the free volume and the molecular volume.

## 4. Conclusions

We have constructed a theoretical approach to deeply understand the alpha and beta relaxation process in amorphous drugs under compression effects. These amorphous drugs are modeled as a fluid of disconnected spheres interacting with each other via the hard-sphere potential. We consider effects of external pressure as a mechanical work done on a single particle. The external work modifies the dynamic free energy, which quantifies interactions of this particle with its nearest neighbors or local dynamics, in the Nonlinear Langevin Equation (NLE) theory. An increase of pressure restricts the local dynamics via enhancing the local barrier height and jump distance obtained in the dynamic free energy. Since the beta JG relaxation in bulk amorphous drugs is motions of a particle within a cage formed by its adjacent spheres, the molecular dynamics of the beta process can be described as NLE calculations for the local dynamics, while the alpha (structural) relaxation time is the mean time when the particle both moves locally and rearranges particles in the first shell to diffuse from its cage. Clearly, the beta and alpha processes are strongly correlated. The particle rearrangement causes the cage dilation and generates the displacement field through a whole space outside the cage. By employing the continuum mechanics analysis, we analytically obtained the displacement field and the collectively elastic barrier to quantify effects of collective motions on the structural relaxation time. Plugging local and elastic barriers into the Kramers’s theory gives the alpha and beta relaxation time at a given pressure.

Based on this approach and the thermal mapping, we quantitatively determined the pressure and temperature dependence of $\tau_{\alpha }$ and $\tau_{\beta }$ of curcumin, glibenclamide, and indomethacin. The thermal mappings for the alpha and beta process are proposed using the thermal expansion in the glassy and crystal state, respectively. The theoretical calculations can be quantitatively comparable with experiments in Refs. [16,17,18]. The quantitative comparisons between theory and experiments clearly validate success and limitations of our approach. In addition, by adjusting the value of $\beta_{c}$ in the thermal mapping to obtain a good agreement with the experimental beta relaxation process, one could estimate the thermal expansion coefficient in the crystal state.
